# Modelling input-output flows of severe acute respiratory syndrome in mainland China

**DOI:** 10.1186/s12889-016-2867-6

**Published:** 2016-02-29

**Authors:** Li Wang, Jinfeng Wang, Chengdong Xu, Tiejun Liu

**Affiliations:** LREIS, Institute of Geographic Sciences and Natural Resources Research, Chinese Academy of Sciences, Beijing, 100101 China; University of Chinese Academy of Sciences, Beijing, 100049 China; Key Laboratory of Surveillance and Early-warning on Infectious Disease, Chinese Center for Disease Control and Prevention, Jiangsu, China; Jiangsu Center for Collaborative Innovation in Geographical Information Resource Development and Application, Jiangsu, China

**Keywords:** Input-output flows, Spatial interaction model, Spatial dependence, Spillover effects, Income difference

## Abstract

**Background:**

Severe acute respiratory syndrome (SARS) originated in China in 2002, and it spread to 26 provinces in mainland China and 32 countries across five continents in a matter of months. This outbreak resulted in 774 deaths. However, the spatial features and potential determinants of SARS input-output flows remain unclear.

**Methods:**

We used an adjusted spatial interaction model to examine the spatial effects and potential factors associated with SARS input-output flows.

**Results:**

The presence of origin-based spatial dependence positively affected SARS input-output flows from the neighbours of the origin regions. Two components of the input-output flows, migrant and hospitalization flows, exhibited distinctive features. The origin-based and destination-based spatial dependence positively affected migrant flows (i.e., due to those seeking jobs) from the neighbours of origin and destination locations. Similarly, the destination-based spatial dependence also positively affected hospitalization flows (i.e., due to those seeking treatment) from the neighbours of destination regions. However, the origin-to-destination based spatial dependence negatively affected hospitalisation flows from the neighbours of origin-to-destination regions. The direct effects accounted for 78 % of the SARS input-output flows, which was 3.56-fold greater than the indirect effects. Differences in regional income drove the SARS input-output flows. Therefore, urban income had a positive effect, whereas rural income had a negative effect. Total interregional flows increased by 3.54 % with a 1 % increase in urban income, and intraregional flows increased by 8.35 %. In contrast, the total interregional flows decreased by 3.38 % with a 1 % increase in rural income, and intraregional flows declined by 2.29 %. Railway capacity, per person gross domestic product (PGDP), urban rate and the law of distance decay also affected the input-output flows.

**Conclusions:**

Our results confirm that the SARS input-output flows presented significant geographic spatial heterogeneity and spatial effects. Income differences were the major cause of the flows between pairs of regions. Railway capacity, PGDP, and urban rate also played important roles. These findings provide valuable information for the Chinese government to control the future spread of nationwide epidemics.

**Electronic supplementary material:**

The online version of this article (doi:10.1186/s12889-016-2867-6) contains supplementary material, which is available to authorized users.

## Background

The increasing movement of people across many regions has tremendous potential to spread infectious diseases [1, 2]. Population mobility enables an infectious disease threat in a single region to become an international dilemma [3]. Mobile populations are closely linked to disease transmission over a wide range. Recent outbreaks of severe acute respiratory syndrome, influenza A virus subtype H5N1, influenza A virus subtype H7N9, Middle East respiratory syndrome, and Ebola haemorrhagic fever were international public health emergencies that elicited widespread public reaction [4–6].

The global spread of SARS passed its 10th anniversary in 2013; however, this outbreak left an indelible mark. The deadly coronavirus erupted in Guangdong in November 2002, and it spread to 26 provinces of mainland China and to 32 countries across five continents in a matter of months and this outbreak resulted in 774 deaths [7]. The short-term economic loss in Singapore, Vietnam, Taiwan, China, and elsewhere in Asia was estimated at $30 billion [8].

Human migration is responsible for the transmission of infectious diseases between humans, and long-range human mobility is a key factor in spatial disease transmission [9–12]. The input and output of SARS cases primarily caused the SARS transmission across the country [7]. SARS was spread across distant locations in China because infected individuals carried the virus when they changed locations, thereby infecting other people in other areas. Long-range human movements were as significant for SARS transmission as direct contact over a close range [13]. The migrant population of mainland China was 261 million in 2010 [14]. Therefore, the potential determinants of infectious disease transmission in this large itinerant population must be investigated.

Identifying how migrant behaviour affects disease dynamics is critical to improve the control efforts of infectious diseases [15]. Three types of models examine how human movement affects the spatial spread of epidemics: the susceptible-infectious-removed, network, and gravity models. The susceptible-infectious-removed model and its extensions incorporate a subdivided population into a migration matrix to describe the movement patterns of individuals within subpopulations [16–18]. Network models incorporate geographic and social distances into topological networks. For example, Xu et al. [19] investigated spatial proximity in epidemic transmission using scale-free networks, and Han et al. [20] examined the effects of human mobility and network topology on the spread of infectious diseases using a hierarchical geographic network. Gravity models are used to capture the spatial features of interregional flows and disease transmission strength with regard to the geographic and economic aspects of epidemiology [21–23].

These epidemic spread models are frequently used to study the factors of infectious disease transmission such as spatial heterogeneity [24], spatial proximity [25], social proximity, and socioeconomic and demographic variables [23, 26]. However, spatial dependence and spatial spillover effects are invariably ignored in the specifications of these models [27]. Accordingly, important characteristics of infectious disease transmission fail to be considered, such as the effects of passing through neighbouring regions and the feedback effects on the source and adjacent areas.

The effect of spatial dependence on disease transmission is commonly measured using spatial models [28–32] including the conditional autoregressive, geographically weighted regression, hierarchical Bayesian, and Moran’s I models. These models measure the effect of spatial dependence on disease transmission using the distance between the origin and the destination. These conventional spatial models apply only to lattice data (not flow data), and they cannot be used to calculate the spatial dependence of origin regions, destination regions, or origin to destination separately.

A typical spatial interaction model (SIM) relies on three types of factors to explain variations in flows: origin-specific, destination-specific, and spatial separation factors [33]. Origin-specific factors reflect the ability to produce flows at the original location (i.e., push factors). Destination-specific factors represent the capacity to attract flows at the destination location (i.e., pull factors). Spatial-separation factors constitute the core of spatial interaction models and reflect the resistance to constraints or act to impede the flows between origin and destination locations. Distance is a typical measurement of the separation between the O-D regions.

The push and pull factors of migration are commonly applied in migration theories. These factors are the driving forces that induce people to move from one location to another. Push factors arise in origin regions and impel people to move away from that location. Pull factors occur in destination regions and attract individuals to that location. People migrate from origin to destination regions for numerous economic, sociopolitical, and environmental reasons such as differences in wages, job opportunities, living conditions, education, and medical care [34–38]. The spread of SARS is a good example of how population mobility facilitates the spread of disease [39].

Spatial interaction models (SIMs) typically explain only variations in interregional flows. However, if intraregional flows are much greater than interregional flows, then this difference adverse effects the local averages of dependent variables [40, 41]. Adjusted SIMs (ASIMs) is developed to avoid this problem [42]. ASIMs use spatial interaction data to explore the driving forces behind the spread of infectious diseases. ASIMs consider the effects of spatial dependence and spillover during epidemic transmission in a heterogeneous environment. This feature addresses the problems with conventional models of epidemic spreading in a homogeneous context.

The direct effects of a spatial homogeneous model refer to the change in a factor within a single region that affects the disease transmission in the region itself. The indirect effects reflect a change in a factor with a single region that potentially affects the disease transmission in all of the other regions [42]. The total effects include both direct and indirect effects.

The present study modelled SARS transmission with an ASIM using input-output flow data to determine its spatial characteristics and potential determinants. We produced input-output flows for SARS based on the spatial location changes of infected individuals in interregional transmission, which reflects the ability of the virus to spread to neighbouring regions [7]. We constructed SARS input-output flows primarily from migrant and hospitalisation flows. Therefore, we focused on these two flow components and the total SARS input-output flows. Control measures for infectious disease often have direct effects on the targeted individuals of one region and indirect effects on the people living in neighbouring regions [43]. The present study focused on the socioeconomic factors and the spatial effects of the SARS input-output flows.

## Methods

### Data

Formal ethical approval was not required because only statistical analyses were applied to the population, and non-human primates were used in the research.

We extracted the SARS input-output flow from 5,327 SARS cases during the outbreak between November 2002 and May 2003 in mainland China. The China Centre for Disease Control provided the data relating to these cases. Each case record in the dataset included the patients’ registered residence, work location or current residence, onset location, reporting units, onset time, and hospitalisation time.

The flow data are a series of limited spatial interactions [44] associated with a pair of origin-destination (O-D) locations representing points or regions in space [45]. These data were used to identify the interactions of individuals’ movements from one location to another within a particular region [41].

**Fig. 1:**
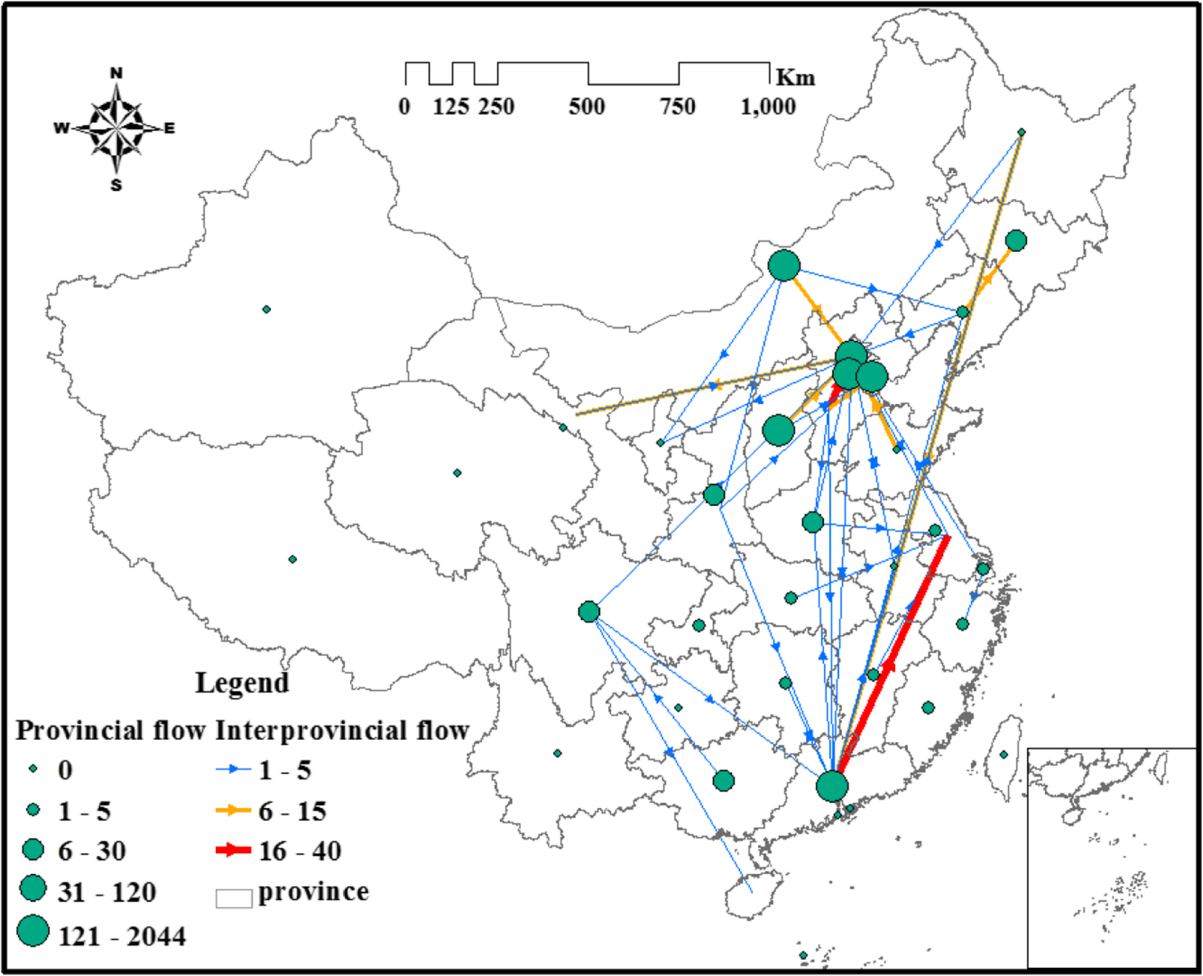
Map of the SARS input-output flows in 2002–03 across mainland China

We produced the SARS input-output flow data for the SARS transmission. We identified individuals from records based on their registered place of residence (*hukou* system) and work location, disease onset location, and location of medical treatment. Figure 1 shows the network of SARS input and output flow in mainland China for 2002–03.

The SARS flow data are composed of intra- and interprovincial flows in the ASIM, each with a pair of origin-to-destination locations. The SARS input-output flow data were formed using an *n*-by-*n* matrix, which represents the SARS flows from *n* regions to each *n* destination (see Additional file 1: Table S2). We divided the SARS input-output flow data into two subpopulations, migrant flow and hospitalisation flow, to determine the social and economic correlates [17] of the interregional spread of SARS. We defined migrant flow as being composed of the individuals who were seeking job opportunities resulting from the difference between the registered residence and work locations. We defined hospitalisation flow as being composed of the individuals who were seeking medical care, which occurred when a difference existed between the disease onset and medical treatment locations. The migrant and hospitalisation flows consisted of intra- and interprovincial flows.

**Fig. 2:**
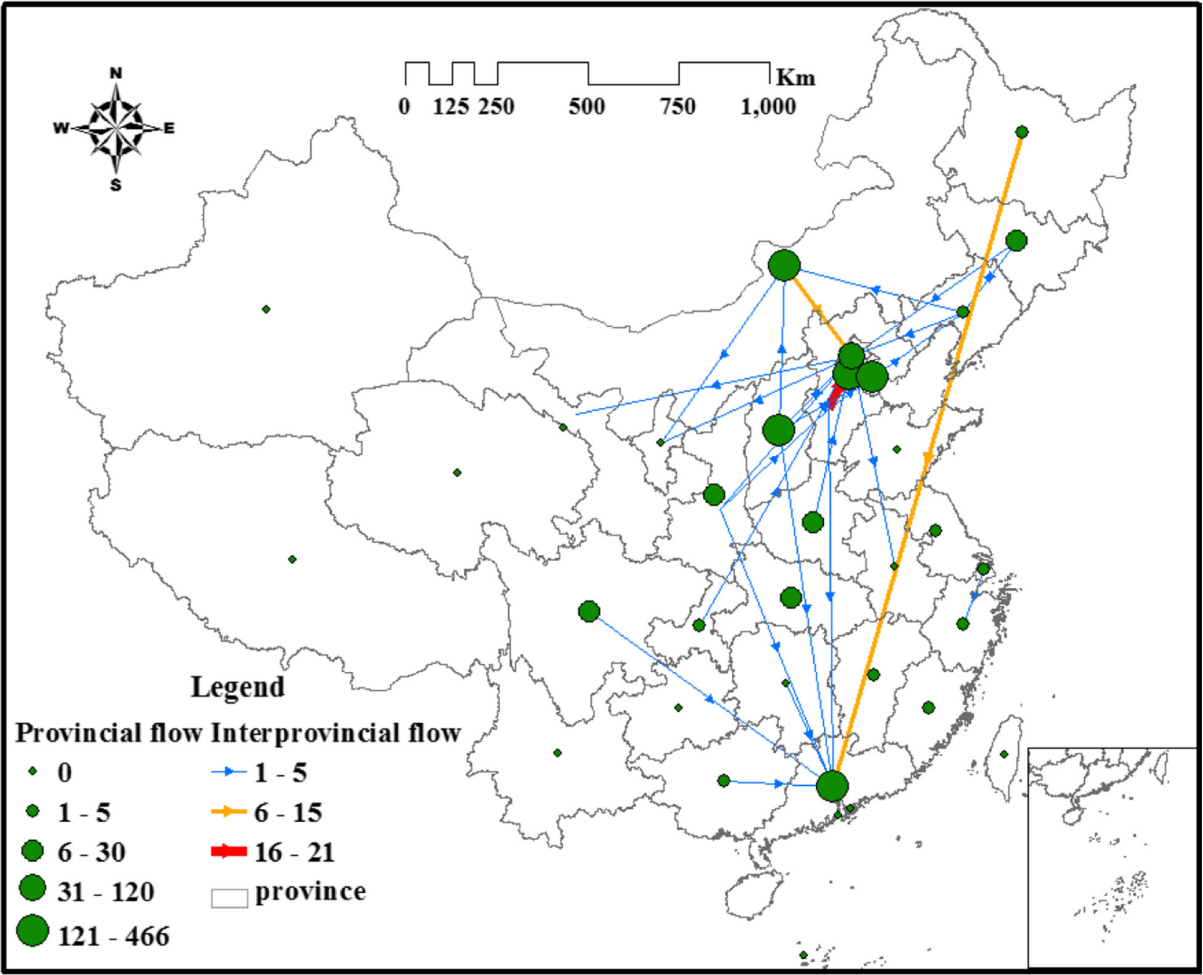
Map of migrant flow in 2002–03 in mainland China

**Fig. 3:**
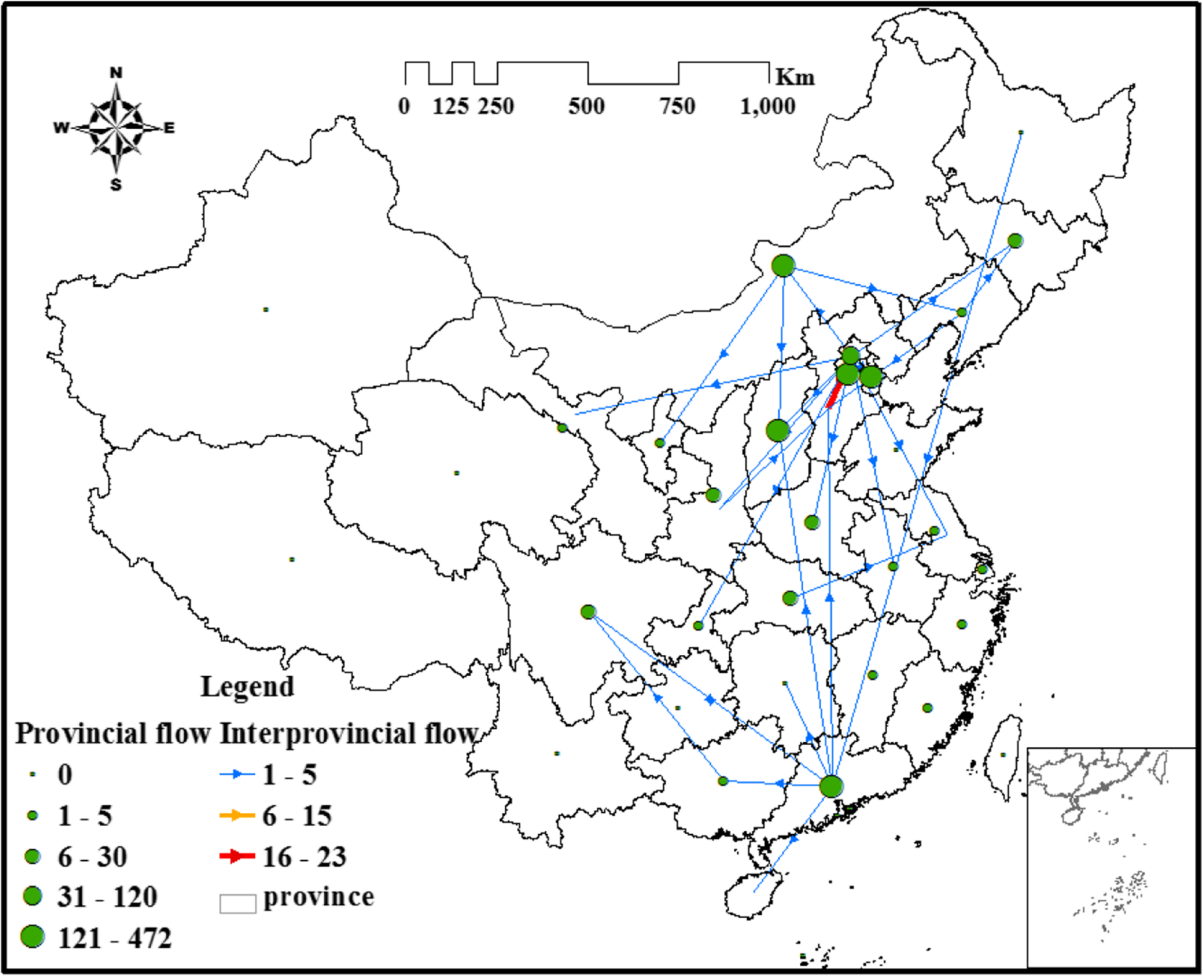
Map of the hospitalisation flow in 2002–03 across mainland China

The generation process of the SARS flow data was presented in a previous study [7]. Briefly, we describe the process of input-output flow extraction. First, two pairs of data records containing spatial location information (e.g., between the registered residence and work location as well as between the onset and medical treatment location) were compared. The record was considered invalid and removed when one of the pairs was missing. Second, geocoding was used to restrict the spatial location information to a provincial or municipal scale. For example, the administrative code of the Haidian District is 110108; however, the higher-level provincial code 110000 was used for location identification. Finally, we identified 1,976 cases in the SARS input-output flows, including 1,491 cases of migration and hospitalisation flows. Figures 2 and 3 show the network of migrant and hospitalisation flows, respectively.

**Fig. 4:**
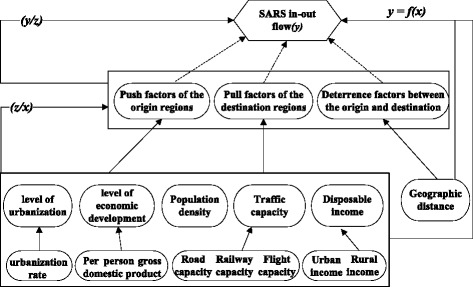
Relationship between SARS input-output flow and its proxy variables

Certain potential factors affect infectious disease transmission in itinerant populations, and this study selected those factors based on the results of previous reports [23, 26, 46–48]. SARS input-output flows during the study period were composed of a mixed population of migrant and hospitalisation flows. Three types of factors affected the magnitude of the flows: push factors of origin; pull factors of destination; and deterrence factors between the origin and destination regions [33, 49]. These factors directly affected the SARS input-output flows, but they could not be quantified. Therefore, we collected a set of corresponding closely related proxies for use for input-output flow modelling. Figure 4 depicts the relationship between the SARS input-output flow and its proxy variables. The urbanization rate, per-person gross domestic product (PGDP), population density, and disposable income were used as proxies for the push or pull factors. We used geographic distance as the proxy for the deterrence factor. Additional file 1: Table S1 summarises and interprets these potential factors. We measured all of the proxies at the provincial or municipal levels. The following section describes the variables.

Urbanization rate signifies the level of urbanization, which indicates the level of economic development, infrastructure, existence of public services, and other related factors. A higher level of urbanization in one region generally indicates a comparative wealth of potential job opportunities and better healthcare, both of which appeal to potential migrants.

PGDP indicates economic development in a country. PGDP is based on the rationale that all residents benefit from their country’s increased economic production.

Higher population densities are often associated with destinations for migrants. For example, several provinces with large populations (e.g., Sichuan, Henan, Shandong, and Anhui) are the most significant source regions for itinerant populations in China. Beijing, Guangdong, and Shanghai are the most important destination municipalities.

Traffic capacity is an accessibility indicator of the transportation network. Road, rail, and air are the three primary means of long- or medium-distance travel in mainland China [50]. Therefore, this study used road, rail, and flight capacity as indicators of total traffic capacity.

Disposable income indicates an individual’s income level. Regional differences produce a growing income gap between the urban and rural areas of mainland China, and higher expected income is a primary cause of migration. This study used the level of disposable income in selected urban and rural areas to determine the effect of income on migration flows.

Geographic distance influences the transportation and psychological costs for migrants. Greater distances involve higher transportation costs, and they weaken social network relationships and the spread of information about destination employment. This study expressed geographic distance as the Euclidean distance between cities.

We obtained socioeconomic data (e.g., urbanization rate, PGDP, population density, and disposable income) from the China Statistical Yearbook for 2004. We derived data relating to road and railway capacity from the Yearbook of China Transportation and Communications for 2004.

### ASIM procedure

SIMs for interregional flows were used to explain the O-D flows between pairs of regions [40]. These models considered the spatial dependence between O-D flows, which more accurately reflects the real world than conventional methods [51]. SIMs are used in many fields of study, including those concerning migration, commodities, information, knowledge, technology, and tourist flows [52–55]. In the field of regional science, the gravity model is also called a spatial interaction model [56], and a log transformation form of the standard gravity model [57] can be expressed as in Eq. 1:

1 $$ y=\alpha {l}_N+{X}_d{\beta}_d+{X}_o{\beta}_o+\gamma g+\varepsilon $$

where *y* is an *N*-by-*1* vector arranged by origin-centric ordering, which is the log transformation of the value of the SARS flows. *N* (*N = n*^*2*^) denotes the number of observations in each O-D pair region. *n* is the number of provinces in mainland China. In this study, *n* = 27 and *N* = 729. *al*_*N*_ is the intercept term. *X*_*o*_ represents the origin characteristics. *X*_*d*_ represents the destination characteristics. These variables capture the interregional variations in the SARS input-output flows. *β*_*o*_ and *β*_*d*_ are the coefficient estimates associated with the origin and destination province characteristics, respectively. *g* signifies the log of the geographic distance between any two provinces in the O-D pairs. The scalar parameter *γ* reflects the effects of distance *g*.

The adjusted spatial interaction model (ASIM) is a new spatial interaction modelling method that uses distance as an explanatory variable and spatial weight matrixes to represent spatial dependence [33]. The ASIM is proposed to determine the potential factors of SARS transmission across space. The equation for the ASIM was expressed as follows:

2 $$ \left({I}_N-{\rho}_o{W}_o\Big)\left({I}_N-{\rho}_d{W}_d\right)y=\alpha {\iota}_N+c{\alpha}_i+{X}_d{\beta}_d+{X}_o{\beta}_o+{X}_i{\beta}_i+\gamma g+\varepsilon \right., $$

where *X*_*i*_ represents the intraregional characteristics and captures intraregional variations in the flows. *I*_*N*_ is an *n*-by-*n* unit matrix. *ca*_*i*_ denotes the interprovincial flow intercept, which constitutes a separate model. *β*_*i*_ describes the coefficient estimates associated with the intraregional characteristics. All of the other parameters in the Eq. (2) are the same as Eq. (1).

Spatial weight matrices provide a convenient way to capture dependency relationships between regions, and we used these matrices to describe the spatial connectivity of the SARS input-output flow. *W* is an *n*-by-*n* nonnegative sparse matrix in the ASIM that describes the spatial connectivity between *n* regions. *W*_*o*_ is an *N*-by-*N* row-standardised spatial weight matrix around each flow origin of all of the destinations using the Kronecker product (*W*_*o*_ = *W* ⊗ *I*_*n*_). *W*_*o*_ captures origin-based dependence, which enhances or diminishes similar flows to neighbouring destinations of the origin. *W*_*d*_ also uses the Kronecker product to produce an *N*-by-*N* spatial weight matrix. *W*_*d*_ captures destination-based dependence (*W*_*d*_ = *I*_*n*_ ⊗ *W*), which enhances or diminishes similar flows to neighbouring destinations. *W*_*w*_ reflects the SARS flow from the neighbours of the origin regions to the destination neighbours. *W*_*w*_ captures the origin-to-destination dependence (*Ww* = *W*_*d*_ ⋅ *W*_*o* =_(*I*_*n*_ ⊗ *W*) ⋅ (*W* ⊗ *I*_*n*_) = *W* ⊗ *W*). *ρ*_*o*_, *ρ*_*d*_, and *ρ*_*w*_ represent spatial dependence parameters associated with the origin-based, destination-based and origin-to-destination-based dependence characteristics, respectively.

The parameters in Eq. (2) can be solved using the maximum likelihood approach, which provides consistent and unbiased results [54]. The form of the log-likelihood function for the spatial flow model specifications appears in the following equation:

3 $$ LnL\left({\rho}_d,{\rho}_o,{\rho}_w\right)=C+ \ln \left|{I}_N-{\rho}_d{W}_d-{\rho}_o{W}_o-{\rho}_w{W}_w\right|-\frac{N}{2} \ln \left(S\left({\rho}_d,{\rho}_o,{\rho}_w\right)\right), $$

where *S*(*ρ*_*d*_, *ρ*_*o*_, *ρ*_*w*_) represents the sum of the squared errors expressed as the scalar dependence parameters alone, and C denotes a constant [40].

These regression coefficients were determined using MATLAB programs developed by our group.

## Results

Our ASIM results (Table 1) indicated a spatial dependence in the SARS input-output flows. We found that the direct effects played a 3.56-fold greater role than the indirect effects. The differences in regional income, urban rate, PGDP, and railway capacity played particularly important roles with regard to the SARS input-output flows. We also confirmed that the law of distance decay exerted an effect. The details of our findings are provided below.

**Table 1:** Total effects of SARS input-output flows estimated using an adjusted spatial econometric interaction model

	SARS input-output flow		Hospitalized flow		Migrant flow	
	Coefficient	*p*-value	Coefficient	*p*-value	Coefficient	*p*-value
Spatial dependence						
ρ_o_	0.201	0.000	0.054	0.218	0.181	0.000
ρ_d_	0.034	0.461	0.171	0.000	0.134	0.002
ρ_w_	0.005	0.947	−0.149	0.032	0.092	0.188
Total effects						
const	−9.176	0.000	−3.984	0.033	−6.844	0.002
ai	−27.053	0.001	−20.852	0.001	−23.875	0.002
o_ Urban rate	−0.172	0.703	0.492	0.134	0.116	0.775
o_PGDP	1.121	0.005	0.364	0.201	0.526	0.125
o_Density	−0.108	0.169	−0.091	0.118	−0.006	0.935
o_Road cap	0.079	0.336	0.074	0.232	0.022	0.760
o_Railway cap	0.235	0.034	0.030	0.702	0.084	0.385
o_Flight cap	−0.104	0.176	0.030	0.594	−0.078	0.258
o_Urban income	2.343	0.002	1.114	0.049	0.740	0.262
o_Rural income	−2.474	0.001	−1.162	0.039	−1.204	0.074
d_ Urban rate	0.816	0.047	0.215	0.471	0.988	0.009
d_PGDP	0.238	0.321	0.176	0.315	0.083	0.696
d_Density	0.003	0.959	−0.010	0.825	−0.067	0.230
d_Road cap	0.168	0.054	0.066	0.299	0.131	0.086
d_Railway cap	−0.093	0.347	0.035	0.628	0.014	0.874
d_Flight cap	−0.060	0.441	−0.117	0.055	−0.112	0.112
d_Urban income	1.197	0.105	0.681	0.221	2.318	0.001
d_Rural income	−0.908	0.141	−0.649	0.159	−1.516	0.007
distance	−0.157	0.116	−0.176	0.015	−0.129	0.165
i_ Urban rate	3.041	0.210	2.372	0.186	3.804	0.087
i_PGDP	−1.181	0.540	−3.206	0.035	1.167	0.495
i_Density	−0.100	0.800	−0.268	0.358	−0.349	0.326
i_Road cap	0.230	0.578	−0.205	0.497	0.533	0.154
i_Railway cap	1.402	0.003	0.787	0.025	1.612	0.000
i_Flight cap	−0.642	0.108	−0.813	0.009	−0.779	0.034
i_Urban income	8.357	0.023	7.424	0.010	7.521	0.023
i_Rural income	−2.290	0.530	1.136	0.675	−5.687	0.085
*R* ^*2*^	0.393		0.523		0.479	
${\overline{R}}^2$	0.371		0.506		0.460	
*log-likelihood*	−160.094		−24.251		−50.304	
Nobs, Nvars	729,	27				

### Spatial dependence and spillover effects

The origin-based spatial dependence generally positively affected the SARS input-output flows from the neighbouring regions of origin locations (*ρ*_*o*_ = 0.201). The two components of input-output flows, migrant and hospitalization flows, presented different features based on the strength of the spatial dependence at different locations. The origin-based and destination-based spatial dependence positively affected migrant flows from the neighbouring origin locations (*ρ*_*o*_ = 0.181) and neighbouring destination locations (*ρ*_*d*_ = 0.134). The destination-based spatial dependence positively affected hospitalisation flows from neighbouring destination regions (*ρ*_*d*_ = 0.171), but a negative effect was observed between regions that neighboured the origin and the destination (*ρ*_*w*_ = −0.149). These results indicate that more hospitalisation flows were produced from origin-to-destination regions and fewer flows arose from the neighbours of origin-to-destination regions.

The direct effects accounted for 78 % of the SARS input-output flows, and the indirect effects (i.e., spatial spillover effects) comprised the remaining 22 % (Additional file 1: Table S4). The direct effects played a 3.56-fold greater role than the indirect effects in the SARS input-output flows.

### Spatial differences in income levels

The differences in regional income played a role in the SARS input-output flows. Urban income exerted a positive effect, and rural income exerted a negative effect. Interregional flows increased 2.34 %, with a 1 % increase in the urban income of the origin regions, and the total intraregional flows increased 8.36 %. In contrast, the total interregional flows decreased 2.47 % with a 1 % increase in the rural income of the origin.

Income differences also played a role with regard to the migrant and hospitalisation flows. A change in the rural income of the destination regions exerted a greater obvious variation in the migrant flow than the hospitalisation flow. In contrast, a change in the urban income of the origin regions caused a greater variation in the hospitalisation flow. The interregional hospitalisation flows increased by 1.11 % with a 1 % increase in the urban income of the origin regions, and the intraregional flows exhibited a 6.6-fold increase over the interregional flows. The interregional migrant flows increased 2.32 % with a 1 % increase in the urban income of the destination regions, and the intraregional flows increased by 7.52 %. However, the interregional hospitalisation flow decreased by 1.16 % with a 1 % increase in the rural income of origin, and the interregional migrant flow decreased by 1.20 %. The intraregional migrant flow exhibited a 3.75-fold increase over the interregional flow.

Other factors also affected the SARS input-output flows. Railway capacity, PGDP of origin, and urban rate of destination had positive effects. The total flow increased by 0.24 % with a 1 % increase in the railway capacity of the origin, and the total intraregional flow exhibited a 6-fold increase. The total flow increased by 1.12 % with a 1 % increase in the PGDP of the origin regions. The total flow increased by 0.82 % with a 1 % increase in the urban rate of the destination regions.

These factors also affected the migrant and hospitalisation flows. For example, regions with a high urban rate received a strong migrant flow. When migration flows to one destination increased by 0.99 % with a 1 % increase in the urban rate in that region, and the regional flows increased by 3.80 %. The PGDP of a region also played a role in the hospitalisation flow. The hospitalisation flow to one region decreased by 3.21 % with a 1 % increase in the PGDP.

### Distance decay

The effect of distance decay was not obvious; however, we confirmed that it affected the SARS input-output flows. Greater geographic distances were associated with reduced SARS input-output flows. The SARS input-output flow decreased by 15.7 % with a 1 % increase in distance. Migrant and hospitalisation flows decreased by 12.9 and 17.6 %, respectively, with a 1 % increase in distance. However, only the distance of the hospitalisation flow was significant at the 5 % level.

## Discussion

This study used an adjusted spatial interaction model to capture the spatial features and potential determinants of inter- and intraregional flows regarding SARS transmission in 2002–03. The model indicated spatial effects on neighbouring regions and identified the potential socioeconomic factors associated with the SARS input-output flows. We found that geographic locations and spatial effects played roles in SARS interregional transmission. We also determined that income differences were key causes of SARS input-output flows between pairs of regions. Railway capacity, PGDP, and urban rate also played important roles.

The gravity model used commuting data to capture the relationship between the movement of people and factors such as geographical distance and population size in disease transmission [21–23, 58]. Some similarities and differences exist between the studies that used the gravity model and the present study, which used an ASIM. The similarities include that diffusive disease spread was related to distance decay. Gravity models successfully capture short-range connections between populations. ASIMs use geographical distance as an explanatory variable and spatial weight matrices to capture spatial dependence. ASIMs capture the short- and long-range connections between different population sizes. We presented the same set of socioeconomic variables associated with the SARS input-output flows in the gravity model for comparison. ASIM simultaneously considered the O-D socioeconomic variables related to intra- and interregional migration flows, whereas the gravity model only considered the socioeconomic variables of the interregional migration flows with regard to the spread of infectious disease.

The gravity model is simpler and often used to explain O-D flows in epidemiology. We used a simple regression model to evaluate each pair of SARS input-output flows to compare the gravity model with the ASIM. Additional file 1: Table S3 presents the results of the SARS flows compared with the gravity model. Table 1 shows that the findings using the ASIM confirmed the relevant spatial effects and socioeconomic factors regarding the spread of SARS. However, the ASIM results have certain advantages. First, the log-likelihood and *R*^*2*^ values are much higher than those of the gravity model. Higher such values indicate better model fits to the data. Second, the results of the ASIM appear to be more realistic than those of the gravity model, and the former explained the estimated coefficients under the 10 % significance level. Third, only the ASIM were able to estimate the factors related to the intraregional flows simultaneously.

Disease transmission is generally characterized by the aggregation or clustering in one location and its neighbouring regions. Therefore, flows from nearby locations are similar in magnitude [27] and measured via the spatial dependence index. The factors that determine disease transmission in a region can also affect neighbouring regions indirectly, and this phenomenon is known as “spillover”. Spatial dependence and spillover are important indices when examining disease cluster and transmission characteristics. The potential effects of spatial dependence and spillover increase the importance of balancing medical resources between providing health services in migrating host regions (e.g., Hebei, Inner Mongolia, Shanxi, Shandong, and Sichuan) and managing infectious diseases in migrant-receiving areas (e.g., Beijing, Guangdong, and Tianjin). Therefore, it is important to address current public health management issues including the education of healthcare professionals, management guidelines for non-endemic areas, and the use of health services and health outcomes in both migrant and local populations.

The income differences between the provinces in mainland China played an important role in the nationwide transmission of SARS. Urban income was shown to always play a positive role; however, rural income often played a negative role. This relationship reflects the ultimate effect of migration: the pursuit of potential job opportunities and expected income. The level of urban income in the origin region was also a pull factor for hospitalisation-related flows. Regions with higher urban incomes attracted more people who were seeking medical treatment, and rural income was a push factor in origin regions such as Hebei, Inner Mongolia, and Heilongjiang. As such, origin regions with higher rural incomes exhibited lower interregional flows for medical treatment. However, areas with higher rural incomes (e.g., Guangdong, Beijing, Shanghai, and Tianjin) attracted increased intraregional flows for medical treatment. These results suggest that policies seeking to improve the income level of rural residents will reduce the role of infected migrants in the interregional transmission of infectious disease.

Our study confirmed that the large-scale spread of infectious diseases such as SARS is related to railway capacity, PGDP, and urban rate. Notably, migrant workers did not have access to healthcare in receiving cities. Thus, migrants returned to their original areas for treatment, which might have consequently facilitated epidemic dissemination. Our findings offer empirical support for the domination of migration motivation via economic mechanisms and the effect of migration patterns on the spatial dynamics of disease dissemination. These results suggest that improving the levels of economic development and rural income is a method to reduce infectious disease transmission as measured by SARS input-output flows.

Migration flows were the primary cause of the spread of SARS from China’s central cities to its remote rural areas. It is necessary for China to promote a nationwide health insurance scheme that can accommodate regional resettlement as soon as possible to reduce the large-scale transmission of another disease outbreak. Hospitalisation flows also led to hospital infections at destination locations. Therefore, it is necessary to balance healthcare resources across the country as well as improve the healthcare and medical infrastructure throughout China’s urbanization.

The spillover effects of the present study suggest that indirect effects arise from the decisions or actions of the neighbourhood [59, 60]. For example, the disease transmission in one region affected the actions of its neighbouring regions. ASIMs allow researchers to separate the effects of the local spillover, which affected handling government resources to address the different characteristics of disease dissemination in the origin, destination regions and their neighbours. This characteristic provides the theoretical basis for local governments to adjust prevention and control strategies as well as the local public budget to address infectious disease emergencies such as SARS.

The uncertainties of this study were primarily due to two aspects: data source and the model employed. The flow data presented in this study were extracted from patients with final diagnoses of SARS. The movement frequencies of the migrant and hospitalisation flows were not obtained for the entire SARS transmission, and this gap represents an area of uncertainty in our analysis. Many potential direct factors were not quantified. Therefore, we used proxy variables, which might represent another source of uncertainty in our data. Another source of uncertainty was the model itself: Spatial relationships in our model were expressed only using their first-order neighbours; however, distant regions might also influence SARS transmission.

## Conclusions

This study found that the SARS input-output flows presented significant geographic spatial heterogeneity and spatial effects. Income differences were a primary cause of the migration flows between pairs of regions. Railway capacity, PGDP, and urban rate also played important roles. These findings illuminate the migration patterns of infected individuals who were motivated by either push or pull factors of spatial features. The results provide valuable information for the Chinese government to control future epidemics.

## Additional file

Additional file 1: Table S1. Description of O-D variables of SARS input-output flows. **Table S2.** SARS input-output flows matrix. **Table S3.** SARS input-output flows estimated using gravity model. **Table S4.** Direct and indirect effects of SARS input-output flows estimated using an ASIM. (DOC 204 kb)

## Abbreviations

SARS: severe acute respiratory syndrome
ASIM: adjusted spatial interaction model
SIMs: spatial interaction models
O-D: origin-destination
PGDP: per-person gross domestic product
