# Supplementary material for: Modelling input-output flows of severe acute respiratory syndrome in mainland China
Source: BMC Public Health. 2016 Feb 29;16:191. doi: 10.1186/s12889-016-2867-6 (PMC4770707; doi:10.1186/s12889-016-2867-6)
Supplement: Additional file 1: Table S1. — Description of O-D variables of SARS input-output flows. Table S2. SARS input-output flows matrix. Table S3. SARS input-output flows estimated using gravity model. Table S4. Direct and indirect effects of SARS input-output flows estimated using an ASIM. (DOC 204 kb) [file 12889_2016_2867_MOESM1_ESM.doc]

**Table S1.** Description of O-D variables of SARS input-output flows

| **Factor Type** | **Chosen Variables** | **Variable Interpretation** |
| --- | --- | --- |
| Origin_variable (o_variable) | o_Urbanization rate(%) | Origin-specific factors that characterize the ability of origin locations to produce or generate migrant flows and reflect interregional variations in the origin of O-D flows |
|  | o_PGDP(yuan per GDP) |  |
|  | o_Density(people per km^2^) |  |
|  | o_Road capacity(million people) |  |
|  | o_Railway capacity(million people) |  |
|  | o_Flight capacity(million people) |  |
|  | o_Urban income(yuan) |  |
|  | o_Rural income(yuan) |  |
| Destination_variable(d_variable) | d_Urbanization rate(%) | Destination-specific factors that represent the attractiveness of destinations and reflect interregional variations in the destination of O-D flows |
|  | d_PGDP(yuan per GDP) |  |
|  | d_Density(people per km^2^) |  |
|  | d_Road capacity(million people) |  |
|  | d_Railway capacity(million people) |  |
|  | d_Flight capacity (million people) |  |
|  | d_Urban income(yuan) |  |
|  | d_Rural income(yuan) |  |
| Internal_variable (i_variable) | i_Urbanization rate(%) | Internal specific factors that capture intraregional variations in flows |
|  | i_PGDP(yuan per GDP) |  |
|  | i_Density(people per km^2^) |  |
|  | i_Road capacity(million people) |  |
|  | i_Railway capacity(million people) |  |
|  | i_Flight capacity (million people) |  |
|  | i_Urban income(yuan) |  |
|  | i_Rural income(yuan) |  |
| Interprovincial | Distance(km) | Origin and destination factors that characterize the way the spatial separation of origin from destination constrains or impedes the interaction |
|  |  |  |

Note: the data of road and railway capacity were obtained from the Year Book of China Transportations & communications 2004 and the other data from the China Statistical Yearbook 2004.

**Table S2.** SARS input-output flows matrix

| **Location** | **Origin** 1 | **Origin** 2 |  | **Origin** *n-1* | **Origin** *n* |
| --- | --- | --- | --- | --- | --- |
| Destination 1 | *o_1_d_1_* | *o_1_d_1_* |  | *o_n-1_d_1_* | *o_n_d_1_* |
| Destination 2 | *o_1_d_2_* | *o_1_d_2_* |  | *o_n-1_d_2_* | *o_n_d_2_* |
|  |  |  |  |  |  |
| Destination *n-*1 | *o_1_d _n-1_* | *o_2_d _n-1_* |  | *o_n-1_d _n-1_* | *o_n_d _n-1_* |
| Destination *n* | *o_1_d_n_* | *o_2_d_n_* |  | *o_n-1_d_n_* | *o_n_d_n_* |

Note: *n is the number of provinces with input or output SARS cases in mainland China; in this study, n=27.*

**Table S3.** SARS input-output flows estimated using gravity model

|  | **SARS input-output flow** | | **Hospitalized flow** | | **Migrant flow** | |
| --- | --- | --- | --- | --- | --- | --- |
|  | **Coefficient** | ***p*-value** | **Coefficient** | ***p*-value** | **Coefficient** | ***p*-value** |
| const | -115.295 | 0.000 | -145.181 | 0.000 | -115.295 | 0.000 |
| o_ Urban rate | -5.296 | 0.848 | -28.145 | 0.077 | -5.296 | -5.296 |
| o_PGDP | 1.719 | 0.744 | -9.385 | 0.030 | 1.719 | 0.744 |
| o_Density | -1.301 | 0.514 | 6.567 | 0.000 | -1.301 | 0.514 |
| o_Road cap | -0.935 | 0.673 | -5.879 | 0.000 | -0.935 | 0.673 |
| o_Railway cap | 0.961 | 0.746 | -5.056 | 0.059 | 0.961 | 0.746 |
| o_Flight cap | -0.671 | 0.437 | -1.750 | 0.078 | -0.671 | 0.437 |
| o_Urban income | 7.823 | 0.678 | 23.221 | 0.011 | 7.823 | 0.678 |
| o_Rural income | 0.503 | 0.952 | 10.775 | 0.236 | 0.504 | 0.952 |
| d_ Urban rate | 6.084 | 0.834 | 26.428 | 0.093 | 6.084 | 0.834 |
| d_PGDP | -1.044 | 0.859 | 6.695 | 0.058 | -1.044 | 0.859 |
| d_Density | 3.178 | 0.138 | -3.387 | 0.038 | 3.178 | 0.138 |
| d_Road cap | -1.944 | 0.376 | 2.701 | 0.075 | -1.949 | 0.376 |
| d_Railway cap | -3.751 | 0.256 | 1.466 | 0.446 | -3.751 | 0.256 |
| d_Flight cap | -2.381 | 0.029 | -1.044 | 0.208 | -2.381 | 0.029 |
| d_Urban income | 25.356 | 0.204 | 5.911 | 0.415 | 25.356 | 0.204 |
| d_Rural income | -16.776 | 0.076 | -15.963 | 0.066 | -16.776 | 0.076 |
| distance | -1.030 | 0.000 | -0.642 | 0.002 | -1.030 | 0.000 |
| ***Pseudo R2*** | 0.256 |  | 0.297 |  | 0.272 |  |
| ***log-likelihood*** | -194.462 |  | -131.694 |  | -140.603 |  |

**Table S4.** Direct and indirect effects of SARS input-output flows estimated using an ASIM

|  | **SARS in-out flow** | | **Hospitalized flow** | | **Migrant flow** | |
| --- | --- | --- | --- | --- | --- | --- |
|  | **Coefficient** | ***p*-value** | **Coefficient** | ***p*-value** | **Coefficient** | ***p*-value** |
| **Direct effects** |  | | | | | |
| const | -7.599 | 0.000 | -3.675 | 0.023 | -5.495 | 0.001 |
| ai | -22.308 | 0.001 | -19.268 | 0.000 | -19.039 | 0.001 |
| o_ Urban rate | -0.140 | 0.705 | 0.461 | 0.131 | 0.091 | 0.775 |
| o_PGDP | 0.929 | 0.004 | 0.339 | 0.196 | 0.425 | 0.120 |
| o_Density | -0.090 | 0.162 | -0.085 | 0.110 | -0.005 | 0.930 |
| o_Road cap | 0.066 | 0.333 | 0.069 | 0.224 | 0.018 | 0.752 |
| o_Railway cap | 0.195 | 0.031 | 0.028 | 0.698 | 0.067 | 0.378 |
| o_Flight cap | -0.086 | 0.171 | 0.027 | 0.597 | -0.062 | 0.249 |
| o_Urban income | 1.941 | 0.002 | 1.036 | 0.043 | 0.594 | 0.255 |
| o_Rural income | -2.052 | 0.001 | -1.084 | 0.036 | -0.969 | 0.070 |
| d_Urban rate | 0.676 | 0.044 | 0.203 | 0.462 | 0.792 | 0.007 |
| d_PGDP | 0.198 | 0.319 | 0.164 | 0.312 | 0.067 | 0.691 |
| d_Density | 0.002 | 0.960 | -0.010 | 0.807 | -0.054 | 0.218 |
| d_Road cap | 0.139 | 0.051 | 0.061 | 0.292 | 0.106 | 0.082 |
| d_Railway cap | -0.076 | 0.344 | 0.033 | 0.623 | 0.012 | 0.868 |
| d_Flight cap | -0.050 | 0.435 | -0.108 | 0.046 | -0.090 | 0.108 |
| d_Urban income | 0.997 | 0.106 | 0.630 | 0.214 | 1.864 | 0.001 |
| d_Rural income | -0.757 | 0.143 | -0.603 | 0.152 | -1.221 | 0.007 |
| distance | -0.133 | 0.123 | -0.168 | 0.026 | -0.104 | 0.163 |
| i_ Urban rate | 2.515 | 0.205 | 2.203 | 0.179 | 3.032 | 0.077 |
| i_PGDP | -0.981 | 0.537 | -2.963 | 0.026 | 0.931 | 0.495 |
| i_Density | -0.082 | 0.800 | -0.248 | 0.354 | -0.279 | 0.317 |
| i_Road cap | 0.190 | 0.574 | -0.193 | 0.487 | 0.428 | 0.142 |
| i_Railway cap | 1.158 | 0.002 | 0.728 | 0.018 | 1.285 | 0.000 |
| i_Flight cap | -0.528 | 0.101 | -0.752 | 0.005 | -0.619 | 0.025 |
| i_Urban income | 6.882 | 0.019 | 6.856 | 0.005 | 5.991 | 0.018 |
| i_Rural income | -1.874 | 0.531 | 1.057 | 0.669 | -4.534 | 0.078 |
| **Indirect effects** | | | | | | |
| const | -1.577 | 0.092 | -0.310 | 0.549 | -1.349 | 0.140 |
| ai | -4.745 | 0.111 | -1.584 | 0.539 | -4.837 | 0.137 |
| o_ Urban rate | -0.032 | 0.724 | 0.031 | 0.685 | 0.025 | 0.796 |
| o_PGDP | 0.192 | 0.115 | 0.025 | 0.644 | 0.101 | 0.286 |
| o_Density | -0.019 | 0.315 | -0.006 | 0.605 | -0.001 | 0.959 |
| o_Road cap | 0.014 | 0.435 | 0.006 | 0.631 | 0.004 | 0.814 |
| o_Railway cap | 0.041 | 0.168 | 0.002 | 0.848 | 0.017 | 0.487 |
| o_Flight cap | -0.018 | 0.314 | 0.003 | 0.732 | -0.016 | 0.394 |
| o_Urban income | 0.403 | 0.113 | 0.078 | 0.600 | 0.146 | 0.404 |
| o_Rural income | -0.422 | 0.099 | -0.078 | 0.607 | -0.235 | 0.239 |
| d_Urban rate | 0.140 | 0.192 | 0.012 | 0.788 | 0.195 | 0.163 |
| d_PGDP | 0.039 | 0.426 | 0.013 | 0.673 | 0.016 | 0.749 |
| d_Density | 0.001 | 0.956 | 0.000 | 0.980 | -0.013 | 0.380 |
| d_Road cap | 0.029 | 0.197 | 0.005 | 0.645 | 0.026 | 0.258 |
| d_Railway cap | -0.017 | 0.434 | 0.002 | 0.830 | 0.002 | 0.906 |
| d_Flight cap | -0.010 | 0.535 | -0.009 | 0.569 | -0.022 | 0.274 |
| d_Urban income | 0.199 | 0.248 | 0.051 | 0.627 | 0.454 | 0.127 |
| d_Rural income | -0.150 | 0.272 | -0.046 | 0.626 | -0.296 | 0.153 |
| distance | -0.025 | 0.237 | -0.008 | 0.725 | -0.025 | 0.314 |
| i_Urba rate | 0.526 | 0.333 | 0.169 | 0.648 | 0.772 | 0.253 |
| i_PGDP | -0.199 | 0.606 | -0.243 | 0.565 | 0.235 | 0.564 |
| i_Density | -0.018 | 0.820 | -0.020 | 0.684 | -0.070 | 0.441 |
| i_Road cap | 0.041 | 0.635 | -0.013 | 0.798 | 0.105 | 0.324 |
| i_Railway cap | 0.245 | 0.119 | 0.059 | 0.564 | 0.326 | 0.123 |
| i_Flight cap | -0.114 | 0.249 | -0.061 | 0.560 | -0.159 | 0.197 |
| i_Urban income | 1.475 | 0.166 | 0.568 | 0.554 | 1.530 | 0.182 |
| i_Rural income | -0.416 | 0.578 | 0.079 | 0.843 | -1.154 | 0.241 |
| log-likelihood | -160.094 | | -24.251 | | -50.304 | |
